# Evolution of European bison image and its implications for current species conservation

**DOI:** 10.1371/journal.pone.0281113

**Published:** 2023-01-31

**Authors:** Tomasz Samojlik, Piotr Daszkiewicz, Anastasia Fedotova, Aurika Ričkienė, Olga Cielemęcka, Marianna Szczygielska

**Affiliations:** 1 Mammal Research Institute, Polish Academy of Sciences, Białowieża, Poland; 2 UMS PatriNat (OFB-CNRS-MNHN), Muséum National D’Histoire Naturelle, Paris, France; 3 Helsinki Collegium for Advanced Studies, University of Helsinki, Helsinki, Finland; 4 Nature Research Centre, Vilnius, Lithuania; 5 Department of Gender Studies, University of Turku, Turku, Finland; 6 Max Planck Institute for the History of Science, Berlin, Germany; Flinders University, AUSTRALIA

## Abstract

Visual media are one of the fastest and most effective tools informing the public about conservation goals and convincing societies to support conservation actions. Similar mechanisms functioned in the past, only within a much longer time scale and different communication channels. We analyse the evolution of European bison’s depictions between 1500 and 1900 in the context of building public awareness of the species and its conservation needs. Experts evaluated the anatomical accuracy of thirty eight images of the species from the period analysed, and their conservation appeal was assessed by using an online survey of the general public. Existing knowledge and previous publications allowed authors to describe the development of the scientific knowledge about European bison in 1500–1900. By juxtaposing this with anatomical accuracy of depictions, a conclusion was reached that the accuracy of depictions was not directly linked to the state of knowledge about the species. In the survey, the public reception of the accuracy of historical pictures of European bison, as well as their potential to be used in conservation campaigns, was connected with subjective appeal of depicted animals. This lesson can be translated to modern conservation campaigns using mass media and global communication channels: popularization of knowledge on species of concern should be accompanied by appealing depictions of these species to strengthen public reception.

## Introduction

We live in a world of global nature decline and biodiversity loss at rates unprecedented in human history: since the 1970s, vertebrate populations have declined by an average of 60% [1], and the remaining mammalian biomass underwent drastic homogenization, with 96% comprised of livestock and humans and only 4% consisting of wild mammal species [2]. An estimated one million plant and animal species are threatened with extinction [3, 4], in large part due to anthropogenic factors: overexploitation, habitat destruction, introduction of non-native species, human-induced climate change. With this, we have entered the phase of Sixth Mass Extinction: the number of species lost in the last century would have been reached in 800 to 10,000 years without human-induced and human-accelerated processes [5]. Slowing down these processes requires global systemic changes on economic, social, political and technological levels, which will not be possible without wide-reaching involvement in conservation [3].

Visual media are nowadays one of the most important channels of human comprehension of information about the world, including political, social and ecological factors. Furthermore, research shows that visual marketing communication tends to be observed as more objective and unmediated capturing of the reality [6]. Scientific illustration is particularly important in this respect, as it is expected to present an “objective” vision of given problem. According to Pyle [7], an illustrator needs to understand the subject depicted which makes communicating the message easier. Apart from direct experience, the natural world is comprehended in the easiest way with the help of visual media, and conversely–all kinds of depictions of nature can affect the way humans perceive, engage in and support conservation efforts. Photographs play an important role in environmental and conservation discourses, as they “give biodiversity a face”, concretize concerns about extinction of species and provide opportunities for affective involvement, which then potentially translates into engagement in wildlife conservation [8]. As authors who analyse environmental discourse observe, it is easier to bypass text than photographs, so images help to capture the attention of the reader/observer [8]. Visual representation of species described in narrative texts strengthens the knowledge acquisition and positively impacts readers’ attitudes [9]. It proves effective in the case of flagship, umbrella and keystone species, where a flagship species acts as a symbol for the protection of a particular habitat, an umbrella species is the one whose protection in conservation of other species or habitats, and keystone species is the one playing such an important role in ecosystem that its loss would impact the entire structure [10]. Exposure to depictions of these relates to conservation intentions and, eventually, engagement in conservation actions [11]. In this respect, concepts such as charismatic or appealing species are crucial for understanding the role of animal imagery in communicating conservation efforts. Charisma of a species is not easily defined [12], and usually is described in traits that charismatic species possess (e.g. beautiful, impressive, endangered; see [13]). Similarly, aesthetic appeal is also expressed in traits, for example large body size, warm and bright colours, anthropomorphic traits and forward-facing eyes [14]. Both of these categories are highly subjective and flexible, nevertheless their importance in conservation was observed [13, 15–18]. Several studies analysed factors influencing public interest in different species and willingness to donate or engage in any form of conservation [14, 19, 20], in general finding connections between public involvement and appealing images. Such images were found effective as promotional tools, not necessarily connected with biodiversity conservation [21, 22]. Animal imagery has the potential to become a major tool for raising social awareness and, consequently, receiving support for animal conservation campaigns. It falls in line with the strategies of conservation marketing that uses methods developed for the commercial sector for the benefit of conservation and is proposed as a fundamental component of modern conservation toolbox [23, 24].

Almost all published studies on the impact of animal imagery on perception, attitudes, and actual engagement in conservation actions embrace only the last decade or two, which makes it difficult to assess the long-term effectiveness of visualization strategies and techniques. An analysis of a case study that is set in the past and which gives an example of an effective action aimed at saving the species from the brink of extinction, could provide a valuable lesson for current species conservation.

In this article, we focused on European bison (*Bison bonasus*), the species that was at the brink of total extinction after being eradicated in Białowieża Primeval Forest (nowadays in Poland and Belarus) in 1919, and was restored by an international effort [25]. European bison, the largest surviving land animal in Europe, is one of the most charismatic and iconic species of European mammals [26]. Its image is widely used in Poland, Lithuania, Belarus, Ukraine and Russia in multiple aspects–as national symbol (emblems of regions, coins, post stamps), as logotype of several institutions and companies, as tourist attraction, as recurring theme on postcards, trinkets and other touristic souvenirs, as branding used in marketing campaigns of different companies, protagonist of children’s books and comics, etc. Especially in Poland, there is little competition for European bison when it comes to the most iconic or charismatic native/local species, attracting the largest interest from the public [27]. Since reintroduction in 1929 until 2020, the population of European bison rose to 9111 individuals worldwide, including 715 in free living population in the Polish part of Białowieża Primeval Forest [28].

The mechanism of building the image of the species (understood as an overall representation, a vision containing both the physical representation and popular knowledge) that requires conservation effort is relatively fast thanks to mass media and social media, wide access to the Internet, and very fast circulation of information [29–31]. It is possible that a similar mechanism also functioned in the past, only within a much longer time scale and different communication channels, with circulation of information restricted to much smaller groups, lower level of knowledge about European bison anatomy among recipients of the information, etc. Many works now explore the relationship between the image of a given species created in various media and the public interest in its status [13, 15, 16, 19, 20, 30], the emotions it evokes in non-specialists [32, 33] and finally the willingness to spend funds on its protection [17, 18, 34]–but all of them focus on recent patterns in a relatively short period of time: from years to a decade. The novelty of our study is that it aims to demonstrate that conservation communication has also been used in the past, except with different channels and over much longer time frame: between 1500 and 1900. The first date is connected with the first known printed images of European bison. The year 1900 was chosen as a boundary date. For the international conservation effort, which started in 1919, to be effective, the process of building an image of European bison likely was completed around this time, especially in the world of slow information circulation. In the 20th century, the role of natural illustrations was gradually taken over by photography, in some aspects considered superior to artistic depiction and more realistic than even the most accurate drawings or paintings [35].

It is possible that the current esteem of the species is the result of a centuries-old process of building the image of European bison as a charismatic, iconic animal. Furthermore, the successful restoration of the species after its extinction in 1919 could have been influenced by this long-lasting process. Previous works [36–38] followed the evolution of naturalists’ knowledge on European bison since the 18th century. In this work, however, we pay attention only to images–although some of them were prepared as book illustrations, they often functioned alone, as prints, leaflets, irrespective of the original book they were published in. By tracing the graphic representations of European bison from 1500 until 1900 we attempt to recreate the evolution of the perception of European bison over the centuries, a process which, we think, could have reflected the development of knowledge about the species, the understanding of its uniqueness and gradual rise of awareness of its possible extinction (particularly in the light of the 19th-century theories of degeneration and inevitable extinction of the species considered as primitive; [39]). Therefore, we hypothesized that: (1) depictions of European bison were becoming more accurate in the course of time between 1500 and 1900, and (2) that the public reception (especially in the context of conservation efforts) of these images was linked with their accuracy.

## Material and methods

Images for this analysis were collected during archival and literature surveys. Historical art collections in Europe were searched for European bison images using online search tools (europeana.eu, polona.pl, artsandculture.google.com, museums.eu), collections’ and museums’ websites, and via direct e-mail inquiries (the latter method was used especially in the case of Polish and Russian collections). The bias of this survey is connected with the level of digitization of collections’ catalogues. Since it is gradually growing, more results should be expected if such enquiry would be repeated in future. The literature search was based on bibliography of several hundred of previously identified publications connected with European bison, Białowieża Primeval Forest and forests of the Grand Duchy of Lithuania published until 1900 in Polish, Russian, English, German and French [38].

During archival and literature surveys also images of red deer *Cervus elaphus* from the same period were obtained (see explanation below).

**Fig 1:**
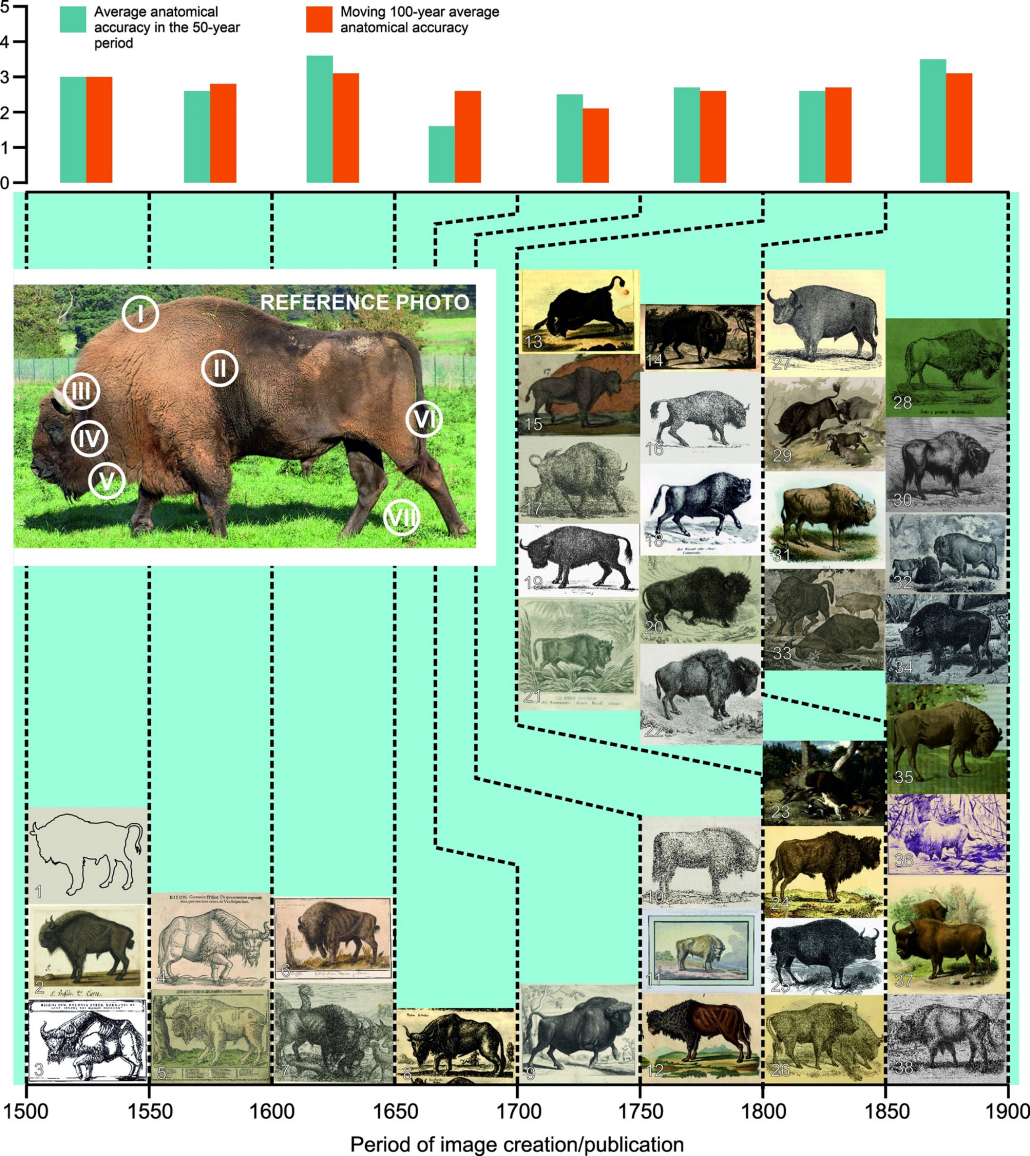
Evaluation of the anatomical accuracy (AA) of historical depictions of European bison based on seven key features of their appearance. (described in Material and methods section). Sources of drawings listed in S1 Appendix.

Around 80 graphical representations of European bison were found, covering the entire analysed timeframe, 1500–1900, out of which 38 were selected for further analysis. Images showing only a small silhouette of the animal were discarded, as well as images that were a clear reprint of previously existing ones (without any added details) and images that, despite description, depicted other species. See the full list of image sources selected for analysis in S1 Appendix–the numbering of the images corresponds to numbering in Fig 1. In case of one image (No 1), we were not able to obtain permission to publish it under CC BY 4.0 license, therefore it is represented by an outline drawn by T. Samojlik (the original image is available for readers to view at the British Museum website–address in S1 Appendix). The analysis of the evolution of graphical depictions of European bison was supplemented by information about the development of knowledge about the species collected from various sources. When considering the knowledge of European bison, the evolution of the methodology of naturalists and their illustrators should be observed. Until the mid-18th century and generation of Linnaeus, the observation of animals as the research method in the field of natural history was rare. In general, scientific observations for the purposes of production of scientific illustrations, especially observation of animals *ad vivum*, from life, was rarely practiced [40].

Initially, 38 archival images were ascribed to 50-year time intervals appropriate to their creation or first publication. Depictions were then analysed for their anatomical correctness. This was done to test the first hypothesis that increasing level of general knowledge about European bison should result in more accurate depictions of the species. The anatomical accuracy (AA) was assessed using seven key features [25, 41] of species’ appearance (identified on a photo from “European bison” entry in Wikipedia, see Fig 1): (I) presence of the hump, gently rising from the neck; (II) body evenly covered with thick fur; (III) horns twisted inwards; (IV) large head set on a strong neck; (V) long hair on chin, throat and front of the body, forming a visible “beard”; (VI) long tail reaching the heel and (VII) overall silhouette: bulky, relatively short, with correct proportions and much heavier front (Fig 1). All historical images were reviewed by five European bison specialists (see Acknowledgments) who gave each image scores based on the presence and correct representation of all these features. Scores (range 0–5) were attributed to each of the seven criteria, and then an average for the entire picture was calculated. This approach was selected as different European bison features were presented on drawings with a varying degree of accuracy. The average score for AA of images from a given interval was calculated (the number of images in one interval ranged from one to fourteen). Additionally, the average score was calculated for the entire century based on average score of respective intervals (given interval and an interval before). The reasoning for that is the assumption that due to slow dissemination of published information in the centuries analysed, both the new images and the ones published in the preceding interval could have equally impacted public perception of European bison (Fig 1).

The second step was to check the perception of historical depictions of European bison. According to our hypothesis, the increasing level of knowledge about European bison should result in more accurate depictions of the species and this should translate to more positive attitudes towards conservation efforts by the general public. Since we have no information how these images were perceived in the time of their creation and distribution, we decided to test the modern public’s evaluation of historical depictions in the context of possible involvement in the conservation of species shown. For this purpose, one image for each interval was selected using the following criteria: (1) images that entered circulation (hand drawn sketches, depictions on manuscripts and paintings that were neither reproduced nor widely known were removed from the pool of images analysed); (2) original compositions; and (3) depictions of a single European bison in similar poses, from the side, with horns clearly visible (so that all images presented in the survey were comparable in terms of composition). The resulting pool of eight images was used to construct a survey containing two questions. The first question was designed to test how viewers assessed the accuracy of the representation in comparison with the reference photo (the same as in experts’ evaluation) with the question: “How accurately does this image depict the European bison (compared to the photograph)?”. The second question was designed to test how the perception of these images would translate to viewers’ involvement in conservation actions. To avoid response bias that could result from asking willingness to pay, about the question focused on the potential of using the given depiction to advocate species protection: “Would you recommend this image for a poster promoting European bison conservation?”. The order of images for the social survey was randomized to remove any chronological context of depictions.

The survey form did not contain requests for any personal information. Participants were only asked to state their professional background, with five possible options: research, nature conservation, forestry, art, other.

An additional part of the survey was designed to check for biases in the modern perception of depictions as old as 500 years: (1) if the illustration style characteristic for given interval was affecting answers; and (2) if the illustrations themselves differed in quality due to the fact that European bison was a species poorly known and one that illustrators had only a slim chance of observing. Red deer was a widely distributed, well-known species whose anatomy should have posed no difficulty to artists throughout the entire interval. When the range of European bison, already rare, was shrinking, red deer was one of the main game animals both in nature and in numerous hunting reserves in Europe. Already in the 17th century red deer antlers, skulls and skeletons were no longer just an ornament but were studier by naturalists [42, 43]. In the same period, William Harvey conducted embryological experiments on hinds, much to the dismay of Valmont de Bomare [44]. The history of this research proves that red deer was easily available, and since the 17th century also its anatomy was no mystery. A control group of depictions of red deer from adequate intervals was therefore added to the survey, with two questions presented for European bison adjusted to red deer: “How accurately does this image depict the red deer (compared to the photograph)?” and “Would you recommend this image for a poster promoting red deer conservation?”.

The survey was distributed online as an anonymous Google form. Information about the survey was posted on social media channels of Mammal Research Institute, Polish Academy of Sciences, as well as distributed via e-mail using authors’ personal contacts. The survey was active online for a period of one month. In total, 145 surveys were collected.

To check the strength of statistical relationships, Pearson correlation tests were conducted between different sets of variables (N = 38 for the first and N = 8 for the rest of the tests):

- time of creation and AA (assessed by experts) of European bison images;
- AA of European bison images evaluated by experts and survey respondents;
- AA of European bison images and their conservation potential (both assessed by survey respondents);
- AA of red deer images and their conservation potential (both assessed by survey respondents);
- AA of European bison images and red deer images in corresponding intervals (both assessed by survey respondents);
- conservation potential of European bison images and red deer images in corresponding intervals (both assessed by survey respondents);
- AA of European bison images assessed by survey respondents with background in science and forestry and others;
- AA of red deer images assessed by survey respondents with background in science and forestry and others;
- conservation potential of European bison images assessed by survey respondents with background in science and forestry and others;
- conservation potential of red deer images assessed by survey respondents with background in science and forestry and others.

## Results

### Anatomical accuracy of historical depictions connected with the development of knowledge about European bison from 1500 to 1900

In the early modern times, the main source of knowledge about European bison anatomy were the works of ancient scholars, mainly Aristotle and Pliny, and sometimes travel accounts, but rarely observations of the actual animal. The interval 1500–1550 was an exception because of two drawings by Albrecht Dürer (Fig 1: 1–2), who had seen the depicted animal (Table 1). Third image from this interval published in “Rerum Moscoviticarum Commentarii” in 1549 was in turn based on vague description (Fig 1: 3, Table 1). However, it was the widely known work of Herberstein that was much more influential than the unknown drawings by Dürer–it was translated, reprinted numerous times and cited [46].

**Table 1:** Historical depictions of European bison with descriptions of their features, corresponding level of knowledge about species and experts’ evaluation of anatomical accuracy (AA) of images. List of analysed works in S1 Appendix.

Time interval	Image No	Description	AA of image evaluated by experts	Mean AA of the interval	Moving average AA of a century
1500–1550	Fig 1: 1	Two drawings by Albrecht Dürer, one of which was accompanied by a description confirming that the artist had seen the depicted animal	3.18	3.0	3.0
	Fig 1: 2		3.68		
	Fig 1: 3	Drawing from the account “Rerum Moscoviticarum Commentarii” by Sigismund von Herberstein, who saw European bison in the Polish-Lithuanian Commonwealth. The anonymous artist of the woodcut most probably relied only on the common knowledge of the European bison	2.21		
1551–1600	Fig 1: 4	Illustration based on the woodcut from Herberstein but including new details (beards, different depiction of fur on the animal’s body)	2.21	2.6	2.8
	Fig 1: 5	Anonymous woodcut printed as a leaflet between 1566 and 1572, depicting European bison caught in Lithuanian woods in 1566 and given to Augustus, Elector of Saxony (1526–1586) for his animal enclosure	3.04		
1601–1650	Fig 1: 6	Watercolour by Anselmus Boëtius de Boodt, part of an album with drawings of quadrupeds commissioned by Emperor Rudolf II around 1600. Despite the fact that official description attributes its creation to the interval 1596–1610, we believe it was made closer to 1608	3.6	3.6	3.1
	Fig 1: 7	Image “Fabel van de bizon en de andere dieren” created by Aegidius Sadeler in 1608 for his collection of woodprints, directly based on de Boodt’s painting, yet flipped horizontally and with some details changed (muzzle proportions, eye positioning)	3.6		
1651–1700	Fig 1: 8	“Bison Iubatus” from Jan Jonston’s “Historiae naturalis de quadrupetibus libri” published in 1655 based on a century-old woodprint from Herberstein’s work with some minor but significant alterations (shape of horns and nasal part of the head, beard, position of the tail)	1.6	1.6	2.6
1701–1750	Fig 1: 9	Cornelis Huyberts’ plate showing European bison published in 1712 edition of Julius Ceasar’s works	2.5	2.5	2.1
1751–1800	Fig 1: 10	Johann Heinrich Müntz’s drawing from circa 1780 of a female European bison kept by naturalist Jean-Emmanuel Gilibert in his mansion in Grodno, near Białowieża Primeval Forest. This image was published in Gilibert’s “Indagatores naturae in Lithuania” in 1781, the work constituting a major breakthrough in the development of knowledge about European bison, and later, in 1805, the same image was made widely popular by Gilibert’s “Abrégé du Système de la nature de Linné”	2.79	2.7	2.6
	Fig 1: 11	Jan Potocki’s watercolour “European bison drawn from nature in Łazienki Park in Warsaw” dated 1792 with, a part of Polish king Stanisław August Poniatowski’s private collection, not widely known	3.82		
	Fig 1: 12	F. J. Bertuch’s depiction of European bison published in 1800 in a series of picture books for children	1.54		
1801–1850	Fig 1: 13	Image of an attacking European bison published in 1807 in Georges-Louis Buffon’s “Natural history. . .” in London was an echo of the breakthrough in knowledge about European bison from the second half of the 18th century, connected with the birth and popularization of Linnaean taxonomy, the work of the French science school, and the creation of “Histoire naturelle. . .”, the first natural history encyclopaedia.	2.93	2.6	2.7
	Fig 1: 14	Hagen’s illustration was far from the species’ real presence, being one of the most curious depictions of European bison’s muzzle as a face	2.36		
	Fig 1: 15	Illustration from Funke’s “The natural history” published in 1820	1.75		
	Fig 1: 16	Kostecki’s depiction of female European bison was probably not widely circulated	3.18		
	Fig 1: 17	Sokołowski’s illustration for Brincken’s widely popular book “Memoire descriptif sur la foret Imperiale de Białowieża” published in 1826	2.79		
	Fig 1: 18	Brodtman’s illustarion published in 1830 in book by Eichwald “Naturhistorische Skizze von Lithauen, Volhynien und Podolien”	3.29		
	Fig 1: 19	Piwarski’s illustration published in 1830 in Jarocki’s book “O Puszczy Białowieskiej i o celniejszych w niej zwierzętach”, most probably based on observation of stuffed animals	3.21		
	Fig 1: 20	Landseer’s image published in 1832	0.68		
	Fig 1: 21	Illustration from Cuvier’s “Oeuvres complètes de Buffon” published in 1835	3.29		
	Fig 1: 22	Auguste’s depiction from the book “La Pologne historique, littéraire, monumentale et pittoresque, ou scènes” by Chodźko, published in 1835–1836	1.14		
	Fig 1: 23	Ruseckas’ painting “Dogs attacking an European bison” from 1843, probably not widely known	3.96		
	Fig 1: 24	Schreber’s illustration from a book published in 1844	2.50		
	Fig 1: 25	An anonymous picture accompanying an article in “Illustrated London News” from 1845	1.71		
	Fig 1: 26	Dolmatov’s illustration from 1849 issue of “The Annals and Magazine of Natural History”	4.18		
	Fig 1: 27	Image by Vasey from “A monograph of the genus Bos. The natural history of bulls, bisons, and buffaloes” from 1857	2.11	3.5	3.1
	Fig 1: 28	Illustration from Dmochowski’s book “Father’s tales on the natural history, geography” from 1859	2.04		
	Fig 1: 29	Zichy’s illustration to a prestigious book published to commemorate tsar’s hunt “Okhota v Belovezhskoi Pushche” frm 1861	3.75		
	Fig 1: 30	Kossak’s illustration to Przybylski’s article published in 1863 in widely popular magazine “Tygodnik Ilustroiwany”	3.64		
	Fig 1: 31	Hengeveld’s illustration from a book published in 1865	3.18		
	Fig 1: 32	Mützel’s depiction of European bison in “Brehms Zoologie” from 1875	3.89		
	Fig 1: 33	Anczyc’s picture from a popular book “Animal life in pictures. European bison” from 1876	3.93		
	Fig 1: 34	Illustration from Brochocki’s article in popular magazine “Wędrowiec” from 1885	4.04		
	Fig 1: 35	Hayek’s illustration in “The great atlas of zoology, botany and mineralogy” published in 1887	4.36		
	Fig 1: 36	Friese’s illustration from 1888, probably with limited ciculation	3.68		
	Fig 1: 37	Lydekker’s depiction from 1898 book “Wild oxen, sheep, & goats of all lands living and extinct”	3.46		
	Fig 1: 38	Beckmann’s illustration with the description “drawn from nature”, before 1900	3.68		

Only two illustrations were found for the interval 1551–1600. The first was based on the woodcut from Herberstein (Fig 1: 4, Table 1), the second was probably inspired by actual observation (Fig 1: 5, Table 1).

In the interval 1601–1650, two depictions of European bison entered circulation: a watercolour by Anselmus Boëtius de Boodt (Fig 1: 6, Table 1) and an image by Aegidius Sadeler (Fig 1:7, Table 1).

There was only one image discovered for the interval 1651–1700: “Bison Iubatus” from Jan Jonston’s “Historiae naturalis de quadrupetibus libri” published in 1655 (Fig 1: 8, Table 1).

Similarly, only one image was ascribed to the interval 1701–1750, Cornelis Huyberts’ plate with European bison published in 1712 (Fig 1:9, Table 1).

There were three images found for the second part of the 18th century, two of which were apparently based on actual observations of live animals: drawing by Johann Heinrich Müntz from around 1780 (Fig 1:10, Table 1), and Jan Potocki’s watercolour “European bison drawn from nature in Łazienki Park in Warsaw” dated 1792 (Fig 1: 11, Table 1). The third image was F. J. Bertuch’s depiction of European bison published in 1800 in a series of picture books for children (Fig 1:12, Table 1).

In the 1801–1850 interval, another milestone for the knowledge on European bison (after Gilibert’s work) was published in 1825: Ludwig Heinrich Bojanus’ “De uro nostrato ejusque sceleto commentatio, *Bovis primigenii* sceleto aucta” [45] (for the discussion about the date of publication see [46]) left a detailed description of the anatomy of European bison. Based on actual observations of animals, rather than ancient descriptions or fragments of pelts or bones, both Gilibert’s and Bojanus’ works can be considered a starting point of the modern research on European bison. Nevertheless, the general level of the knowledge about European bison in the beginning of the 19th century was still limited: publications describing the species were still based on ancient authors rather than on modern observations (e.g. [47]) and despite evidence that European bison and aurochs were separate species, the vigorous debate arguing that they are the same species lasted until the second half of the century.

The number of publications describing or at least mentioning European bison rapidly rose, and the images of the species became more available. There were fourteen images of European bison selected for the analysis for the interval 1801–1850 (Fig 1: 13–26).

Several illustrations were based either on observation of live animals or on specimens from Białowieża Primeval Forest sent to almost all major universities and zoological collections in Europe (for more on this subject see [48] and did not repeat errors characteristic of images from previous intervals, instead highlighting the most important anatomical traits of the species. Nevertheless, there were still publications containing images far from the species’ real presence (Table 1).

The last interval analysed, 1851–1900, was abundant in publications popularizing knowledge on European bison, especially connected with several live animals and specimens sent to several destinations throughout the entire Europe [49]. The idea of degeneration of the species and its inevitable extinction was promoted [39], while others stressed the threatened status of the species [50, 51]. Also in this interval, several original and highly accurate, in terms of anatomical correctness, depictions of European bison were published (Fig 1:27–35, Table 1).

In general, the hypothesis that the gradual rise of knowledge about European bison should result in more precise and anatomically correct depictions of the species in the course of time was not confirmed. No correlation between time of creation and AA of European bison images in consecutive periods evaluated by experts was found (*R* = 0.07, p = 0.8). Chronologically older images were ranked higher or at the same level than more recent ones, with the exception of the last interval, which was assessed the best in terms of AA.

### Modern evaluation of historical depictions of European bison

Among 145 collected surveys, 75 (52%) were filled by persons with the background in science, 15 with experience in nature protection (10%), 20 –in art (14%), 6 –in forestry (4%), and 29 with “other” professional background (20%).

Images of European bison used in the survey (one for each interval) were the ones that were most highly evaluated by experts: their AA was assessed at 2.21 for interval 1501–1550, 3.04 for 1551–1600, 3.57 for 1601–1650, 1.64 for 1651–1700, 2.5 for 1701–1750, 2.79 for 1751–1800, 4.18 for 1801–1850 and 3.64 for 1851–1900. Experts’ assessments of AA of respective images were positively correlated with survey respondents’ evaluation of accuracy (*R* = 0.75, p = 0.02).

**Fig 2:**
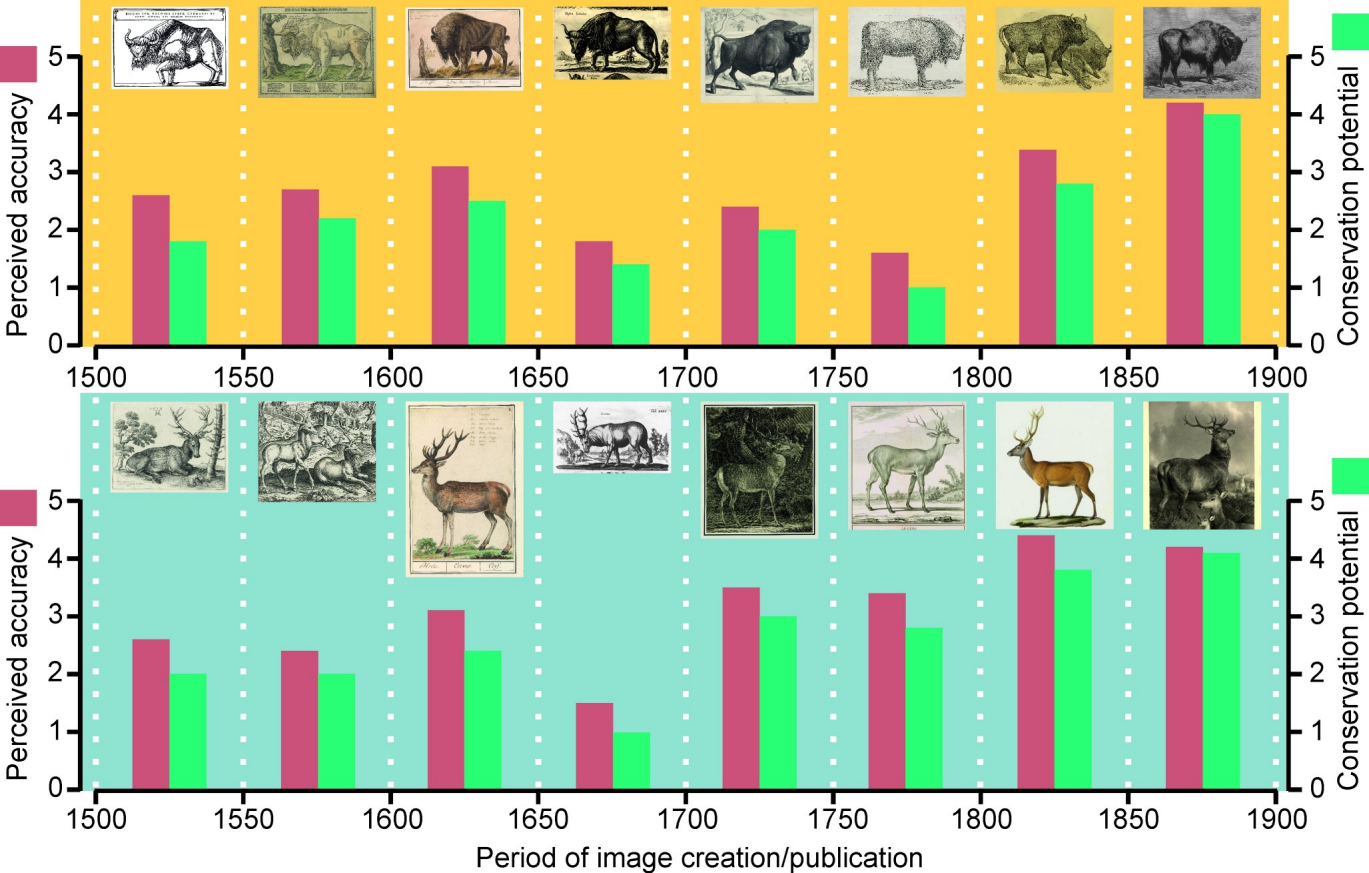
Evaluation of modern perception of anatomical accuracy and conservation potential of historical depictions of European bison (above) and red deer (below).

In general, the potential of using images in conservation of European bison was ranked lower than the perceived correctness of depictions (Fig 2). The same phenomenon was observed in the case of depictions of red deer–there was not even a single case where conservation potential would be assessed at a higher level than the accuracy of depiction. There was a high correlation between perceived correctness of the image and its conservation appeal in the case of both species: *R* = 0.98, p<0.05 in the case of European bison and *R* = 0.99, p<0.05 in the case of red deer.

A low bias was found in the modern perception of historical depictions connected with illustration style characteristic for each interval and possible lower quality of illustrations due to slim chance of observing live European bison by illustrators: there was a high correlation between the assessment of the accuracy of depictions of European bison and deer in corresponding intervals (*R* = 0.7, p = 0.04) and between the conservation potential of images of both species (*R* = 0.8, p = 0.01).

To check if there was difference between answers in survey forms from respondents with background in science and forestry and the rest, we checked the correlation between AA assessment of European bison images in these two groups (*R* = 0.98, p<0.05), between AA assessment of red deer images (*R* = 1.0, p<0.05), between conservation potential of European bison images (*R* = 0.92, p<0.05) and between conservation potential of red deer images (*R* = 1.0, p<0.05).

Our hypothesis that the contemporary public reception of European bison images (both in observers’ evaluation of the correctness of depictions and their conservation potential) would be linked with the period of creation was not confirmed. Instead, the modern perception of anatomical accuracy was nonchronological. Images from two last intervals were evaluated higher in both aspects, in line with experts’ assessment. Conservation potential of images assessed by viewers was strongly linked with their evaluation of accuracy of depictions.

## Discussion

Discussing the development of knowledge about European bison, several circumstances should be considered. First, the gradual decrease of the geographical range of European bison since early mediaeval times made it difficult for naturalists to observe the animal at all, all the more in its natural habitat. The European bison was often confused with another bovine–aurochs *Bos primigenius* [52, 53]. Taxonomic debates whether European bison and aurochs were separate or the same species lasted until the 19th century. It was understandable, given the low level of knowledge on aurochs–its last surviving population went extinct in 1627 in Jaktorów in east-central Poland, where aurochs were protected by the Polish kings [54]. The species was not widely known to Western European naturalists. What is more, in the mid-18th century even the possibility that European bison belonged to the Scottish cattle breeds was considered. The confusion was increased by first reports on American bison *Bison bison* written by travellers since the 16th century [55]. The development of scientific knowledge about European bison was traced in a series of books [36–38], but the local, traditional knowledge about the species in the period concerned remains largely unknown. The only glimpse to traditional knowledge we have is through descriptions of customs from the Grand Duchy of Lithuania (in forests of which European bison survived the longest). In 1582, Maciej Stryjkowski noted that Lithuanians used European bison pelts to build boats [56]. In 1781, Jean Emmanuel Gilibert observed that it was an old Lithuanian custom to use pieces of fur from European bison’s forehead as the remedy for difficult births [57]. In 1846, Ludwik Jucewicz wrote about another “magical” meaning ascribed to European bison: amulets and shields were made from their pelts [58].

Our study has shown that the centuries-long process of shifting the status of European bison from mythical beast connected with local folklore to an actual species in need of conservation, along with building the knowledge base on European bison and creating the idea of European bison as a charismatic, iconic animal had no immediate effect on the correctness of depictions of the species. Anatomical accuracy of depictions was most probably linked more with the direct observation of the animal by artists and art style of given period than with the existing knowledge about the species at the time. The 19th century–the period with the most rapid development of scientific and popular knowledge about European bison, was also the period most abundant with anatomically accurate images of the species. The modern perception of correctness of historical depictions mostly followed experts’ evaluation and was also nonchronological. Despite being subjective, the perceived correctness of depictions was linked with modern assessment of conservation appeal of historical images of European bison.

The novelty of this study which is the ability to reach back with the analysis to the year 1500 and observe the development of images depicting species nowadays considered iconic is also its biggest constraint. There is no way to test the actual influence of the imagery on the perception of the species, as well as the direct impact of popularization of knowledge and widespread use of European bison images on the success of the recovery campaign after the extinction of the species in the wild in 1919. We are aware that the perception of natural world was different in previous ages and changing through time. The example of such changing perception is the female beauty canon in European art [59], and obviously current awareness of nature conservation is very different than in eras when the concept of conservation itself was non-existent. Our decision to test the modern public’s evaluation of historical depictions and resulting analysis is nevertheless justified when it comes to the lessons for contemporary conservation campaigns. It is the modern perspective they must appeal to when using imagery of European bison or other species. What we know for a fact is that the International Society for the Protection of European Bison used historical images and photographs of European bison in their publications to promote the idea of species conservation, e.g. in the album published after the 5th annual meeting of the society in 1929 [60], which falls after the scope of this work. Perhaps the historical imagery was believed to carry a stronger message in building the status of European bison as an “ancient beast”. Another potential effect of this “ancient beast” status is the fear or general negative attitude that this species induces in people (especially in areas where it does not occur) [61]. Measuring the impact of such imagery would be much easier today, achievable through direct information on donations transferred by individuals to organizations responsible for conservation campaigns around particular species. Nevertheless, our study allows us to draw some lessons from the past, as it falls in line with recent research on pro-environmental outcomes of using animal imagery.

First, it seems that the aesthetic appeal of animal imagery has proven to have an impact on attitudes towards wildlife conservation. In a review study [62] images of animals were found to have positive effects on emotional responses in people and their willingness to contribute to animal conservation, but it was not the anatomic accuracy of pictures but rather aesthetic appeal of the image and amount of exposure that contributed to these impacts. The use of carefully selected visual material in mass media can change people’s attitudes and behaviours [32], and photographs of animals are a powerful tool of such campaigns. This is observed particularly in social media which are a new and rapidly developing tool for increasing public awareness about endangered species. When articles posted on social media are accompanied by abundant high-quality images, videos and animations [30], it can foster kinship with animals and respect for their sentience and individuality in observers [33]. Other study [18] found that the level of public support was higher for so-called appealing or charismatic species. The appeal or charisma of a species is a complex variable, consisting of traits such as the size of an animal (with bigger animals being usually more attractive; [16]) or presence of forward-facing eyes [17]. European bison fits both these categories.

Second, what our study showed was the fact that the actual anatomical correctness of images plays a secondary role to subjective perception, which was particularly visible in two intervals of the 19th century, where experts’ evaluation did not match the one from public surveys. Such subjective view of is also observed today, to an extent that even cartoony versions of animals (albeit carefully selected) can have positive impact on observers’ attitudes towards conservation campaigns [63]. Presenting animals in animation is seen as a tool of building charisma of species [13] but also influences public interest and increases conservation efforts, such as donations for zoo animals [20]. Animated animal-focused movies affected public awareness about featured species (e.g., fossa *Cryptoprocta ferox* featured in the movie “Madagascar”, Spix’s macaw *Cyanopsitta spixii* in”Rio” and blue tang *Paracanthurus hepatus* in “Finding Dory”) [19].

Although two thirds of surveys came from persons with background in science or forestry, who were potentially more invested in species conservation and possessed higher level of knowledge about European bison, no difference between their answers and answers from persons with different backgrounds were found. This suggests that education and professional background of surveyed persons did not bias the results of the study.

But there are some constraints of using images of iconic animals in conservation campaigns. Almost 200 threatened species that fit the category of large species with forward-facing eyes were identified as not widely used for conservation campaigns [17]. This was explained by the fact that conservation campaigns tend to be overly conservative and focus only on a few well-known, large and aesthetically appealing mammals, overlooking many “Cinderella” species. This falls in line with another study [34] observing that donations in conservation programmes not necessarily echoed the endangered species status, being rather driven by the charisma of the species (or even order of presentation on website, [18]) than ecological or scientific considerations. Flagship species used as a conservation organizations’ marketing tool to promote and encourage public support for preventing the loss of biodiversity were also selected for their perceived charisma [22]. In some cases such species often serve as logos, emblems, or marketing visual symbols used to promote consumer products as detached from wildlife as alcohol [21]. It was suggested [64] that shifting attention between charismatic and unpopular species can be achieved by directing the marketing effort to specific groups, and also by delivering additional information about less appealing species, as it has been done for bats in the wake of white-nose syndrome in North America [65]. An evidence was provided [66] that in general, people value rare species more than they do common species which can potentially have negative outcomes such as exploitation (e.g. ecotourism or exotic pet trade, see [67]) of rare and endangered species leading to their extinction. Clucas *et al*. [16] show that conservation organizations tend to focus their publicity on large charismatic species and in this way end up communicating a selected and narrow sample of conservation problems. A paradox was pointed out: the most charismatic species are at risk of imminent extinction while their media presence is strong [68]. Species often seen in media can be easily and falsely perceived as abundant in nature, which leads to lack of public awareness of the actual, threatened status of these species. Even in the most drastic cases when a species is presented as on the brink of extinction, this wide media coverage raises the public awareness only for a limited period of time, after which the event is no longer perceived as noteworthy [69].

In the case of European bison the long-lasting building of the status of an iconic species, accompanied by knowledge accumulation and presentation of the animal in rising numbers of depictions could have contributed to a positive outcome of restoration initiatives after species extinction in the wild in 1919. International collaboration brought the animal back to BPF in 1929 where it is thriving now. Despite centuries dividing our era and the times discussed in this article, the general mechanism of building up a vision of European bison is the same–popularizing the increasing knowledge base and presenting depictions of the species, with different levels of anatomical accuracy but focusing more on appeal to the viewer. With modern media and unprecedented speed of global communication, now is the chance to employ similar mechanisms to promote conservation campaigns and build appealing images of other species. Less charismatic, but not less important for biodiversity conservation.

## Conclusions

1. Contemporary status of European bison as an iconic species was built in a centuries-long process involving development of knowledge and evolution of depictions of the species.
2. Apart from the 19th century, when the knowledge base on the species increased, the accuracy of depictions did not reflect the state of knowledge.
3. The perceived conservation potential of historical images assessed by modern viewers was strongly linked with their evaluation of accuracy of depictions.
4. Despite differences in available means of communication and general knowledge, mechanisms behind building up a vision of European bison were similar to contemporary means.
5. Modern media and speed of global communication offer an opportunity to use similar mechanisms: popularizing the knowledge and presenting appealing depictions of species should also be used in conservation efforts.

## Supporting information

S1 Appendix List of historical illustration sources (listed in the order of appearance on Fig 1). (DOCX)

S1 File (PDF)

S2 File (PDF)

S3 File (DOCX)
