# Supplementary material for: Evolution of European bison image and its implications for current species conservation
Source: PLoS One. 2023 Jan 31;18(1):e0281113. doi: 10.1371/journal.pone.0281113 (PMC9888683; doi:10.1371/journal.pone.0281113)
Supplement: S1 Appendix — (DOCX) [file pone.0281113.s001.docx]

**Appendix 1. List of historical illustration sources (listed in the order of appearance on Fig.1).**

1. Outline based on the drawing by Dürer A (ca 1501-1504) drawn by Tomasz Samojlik.

The original image: Dürer Albrecht (ca 1501-1504) Verso: European bison; standing in profile to l, looking to front Pen and black ink. The British Museum, SL,5261.1 to 167 is available online at www.britishmuseum.org/collection/object/P_SL-5261-101

2. Dürer A (before 1528) Sheet with a Bison. Lombard album (ca 1550-ca 1570), RP-T-1952-351, Rijksmuseum (http://hdl.handle.net/10934/RM0001.COLLECT.29680)

3. Herberstein Siegmund von (1549) Rerum Moscoviticarum Commentarii. Wien.

4. Gessner C (1554) Historiæ animalivm. Zurich.

5. Anonymous (1566-1572) Diß ist deß Wilden Awerochsen Contrafactur. Zentralbibliothek Zürich, Graphische Sammlung und Fotoarchiv: Aus der Sammlung von Johann Jakob Wick (Shelf Mark: PAS II 9/2; dx.doi.org/10.7891/e-manuscripta-92207)

6. Sadeler A (1608) Fabel van de bizon en de andere dieren, from: Theatrum morum; artliche Gesprach der Thier mit wahren Historien den Menschen zur Lehr, RP-P-OB-5210, Rijksmuseum (http://hdl.handle.net/10934/RM0001.COLLECT.168100)

7. de Boodt AB (1596-1610/around 1608) Wisent. RP-T-BR-2017-1-2-15, Rijksmuseum (http://hdl.handle.net/10934/RM0001.COLLECT.673390)

8. Jonston J (1655) Bison Iubatus. Historiae naturalis de quadrupetibus libri. Amsterdam (https://polona.pl/item/historiae-naturalis-de-quadrupedibus-libri-cum-aeneis-figuris,MTg1NDM1NDg/80/#item)

9. Huyberts C (1712) Bizon. RP-P-OB-67.749, Rijksmuseum (http://hdl.handle.net/10934/RM0001.COLLECT.336499)

10. Müntz JH (c. 1780) Female European bison of Jean-Emmanuel Gilibert, in: Gilibert J-E (1781) Indagatores naturae in Lithuania. Vilnius and Gilibert J-E (1805) Abrégé du Système de la nature de Linné, histoire des mammaires, ou des quadrupèdes et cétacées. Lyon.

11. Potocki J (1792) Żubr narysowany z natury w Łazienkach Warszawskich (European bison drawn from nature in Łazienki Park in Warsaw). Department of Drawings of Warsaw University Library, Royal Collection, Inw. zb. d. 10206.

12. Bertuch FJ (1800) Vierfüßige Tiere, Taffel XXVII. Bilderbuch für Kinder, Weimar (https://doi.org/10.11588/diglit.2630).

13. Buffon G-LL de (1807) Buffon’s Natural history, containing a theory of the earth, a general history of man, of the brute creation, and of vegetables, minerals, etc. Vol. VIII, Printed for the Proprietor, London (https://doi.org/10.5962/bhl.title.36928).

14. Hagen KG (1819) Geschichte des Preußischen Auers nebst einer Abbildung desselben. In: Beiträge zur Kunde Preußens 2: 206–234 (https://dlibra.bibliotekaelblaska.pl/dlibra/publication/48096/edition/45288/content).

15. Funke KP (1820) Historya naturalna (The natural history). Wrocław (https://www.sbc.org.pl/dlibra/publication/93016/edition/87770/content)

16. Kostecki F (1820-1829) Żubrzyca (Female European bison). Biblioteka Narodowa, Magazyn Ikonografii G.11325/II (https://polona.pl/item/zubrzyca,MTE5MTU2Mzkz).

17. Sokołowski J (c. 1821) Żubr (European bison). In: Brincken J (1826) Memoire descriptif sur la foret Imperiale de Białowieża, Warszawa (https://www.biodiversitylibrary.org/bibliography/70897).

18. Brodtman (1825) European bison. In: Eichwald EK (1830) Naturhistorische Skizze von Lithauen, Volhynien und Podolien. Vilnius (https://books.google.pl/books?id=_3VNAAAAcAAJ).

19. Piwarski JF (1830) Żubr z natury (European bison from nature). In: Jarocki FP (1830) O Puszczy Białowieskiej i o celniejszych w niej zwierzętach (On Białowieża Forest and its most notable animals), Warszawa.

20. Landseer T (1832) The Bonassus. In: Barlow JH (1832) From Characteristic sketches of animals, principally in the Zoological Garden, Regents Park. London (https://books.google.pl/books?id=__heAAAAcAAJ).

21. Cuvier G (1835) Oeuvres complètes de Buffon. Vol. 5, Paris (https://www.biodiversitylibrary.org/item/198514#page/9/mode/1up).

22. Auguste FA (1835-1836) La Forêt de Bialowiez - Le Bison, L'Urus. In: Chodźko L (1835-1836) La Pologne historique, littéraire, monumentale et pittoresque, ou scènes historiques. Vol. 1, Bureau Central, Paris (https://www.dbc.wroc.pl/dlibra/publication/6508/edition/6031?language=en).

23. Ruseckas K (1843) Dogs attacking an European bison. Lithuanian National Museum of Art T-1498.

24. Schreber JCD (1844) Die Säugthiere in Abbildungen nach der Natur mit Beschreibungen (Tafelband 2): Theil 4 - 7 und Supplement 3 u. 4: Taf. CCXCV (https://digi.ub.uni-heidelberg.de/diglit/schreber1844tafelbd2/0272/image)

25. Anonymous (1845) European bison. At the British Museum. Illustrated London News 180(7): 237 (https://books.google.pl/books?id=7q5LAAAAcAAJ).

26. Dolmatov D (1849) Note on the capture of aurochs (Bos urus, Bodd). The Annals and Magazine of Natural History 3: 148 (https://www.biodiversitylibrary.org/item/54554#page/161/mode/1up).

27. Vasey G (1857) A monograph of the genus Bos. The natural history of bulls, bisons, and buffaloes. John Russel Smith, London: 40 (https://www.biodiversitylibrary.org/item/5638).

28. Dmochowski FJ (1859) Opowiadania ojca obejmujące historyą naturalną, jeografią, historyą polską i starożytną, powieści i poezye (Father’s tales on the natural history, geography, Polish and ancient history, storeis and poems). Vol 2. Drukarnia J. Jaworskiego, Warszawa (https://polona.pl/item/opowiadania-ojca-obejmujace-historya-naturalna-jeografia-historya-polska-i-starozytna,MTA3MTY2NA/135/#item).

29. Zichy M (1862) Zubr (European bison). In: Fuchs V, Zichy M (1862) Okhota v Belovezhskoi Pushche. St. Petersburg.

30. Kossak J (1863) Żubr. In: Przybylski W (1863) Notatki z wycieczek po kraju. Puszcza Białowieska i żubry. Tygodnik Ilustrowany 200: 289 (https://bcul.lib.uni.lodz.pl/dlibra/publication/1512/edition/1174/content).

31. Hengeveld GL (1865) Europesche Bisonstier. In: Hagenveld GL (1865) Het rundvee, zijne verschillende soorten, rassen en veredeling. De Erven Loosjes, Haarlem (https://books.google.pl/books?id=H1NVAAAAcAAJ).

32. Mützel G (1875) Wisent. In: Brehms Zoologie, 1. Abt. Leipzig (https://www.biodiversitylibrary.org/item/16032#page/424/mode/1up).

33. Anczyc LW (1876) Obrazki z życia zwierząt. Żubr (Animal life in pictures. European bison). Czytelnia Ludowa, Kraków (https://polona.pl/item/obrazki-z-zycia-zwierzat-bobr,MzkzOTYyNDY/97/#item).

34. Brochocki W (1885) Z Puszczy Białowieskiej: żubr (From Białowieża Primeval Forest: European bison). Wędrowiec 23(38): 450 (https://bcul.lib.uni.lodz.pl/dlibra/publication/421/edition/272/content).

35. Hayek G (1887) Wielki atlas do zoologii, botaniki i mineralogii (The great atlas of zoology, botany and mineralogy). Warszawa (https://kpbc.umk.pl/dlibra/publication/30919/edition/39801/content).

36. Friese R (1888) Wisent. Collectie Rijksmuseum Twenthe, Enschede. Bruikleen particuliere collectie, Haaksbergen, inv. BR3092 (https://collectie.rijksmuseumtwenthe.nl/zoeken-in-de-collectie/detail/id/2dc5dfd6-e1db-55c8-bc3a-8ba016817df7).

37. Lydekker R (1898) Wild oxen, sheep, & goats of all lands living and extinct. Rowland Ward, London, plate V (https://www.biodiversitylibrary.org/item/36741#page/97/mode/1up).

38. Beckmann L (before 1900) Die Auerstiere im Zoologischen Garten zu Köln. Nach der Natur gezeichnet von L. Beckmann. National Museum in Warsaw, nr inw. Gr.Pol.28499.
