# Supplementary material for: Evolution of European bison image and its implications for current species conservation
Source: PLoS One. 2023 Jan 31;18(1):e0281113. doi: 10.1371/journal.pone.0281113 (PMC9888683; doi:10.1371/journal.pone.0281113)
Supplement: S1 File — (PDF) [file pone.0281113.s002.pdf]

Request for Permission to Publish Content under CC-BY License

Dear Rights Holder or Representative,

I have submitted a paper for publication in a PLOS journal, and wish to include the content listed below in the paper. I'm hereby requesting your (or your company's or institution's) permission to include the content in my paper. Please note that all PLOS journals are published under a Creative Commons Attribution License (CC BY), which allows for unrestricted use and distribution, even commercial, as long as attribution is given to the creator or rights holder of the content. See https://creativecommons.org/licenses/by/4.0/.

To grant me permission to use the content in my PLOS paper, please fill in the information below and then scan the completed form and send it to me at my email address.

Thank you.

My name:

Tomasz Samojlik

My email address:

samojlik@ibs.bialowieza.pl

Description of the content which I'm seeking permission to use (citation and/or title, and pasted screen shot, if applicable):

L. Beckmann, "Żubr w zagrodzie", Gr. Pol. 28499

Link to the Content:

* * *

On behalf of myself or the rights holder, I hereby grant the permission sought herein.

Signature of Party Granting Permission:

National Museum in Warsaw

Date:

02.06.2022

Printed Name and Title:

Kierownik
Działu Dokumentacji Wizualnej i Digitalizacji
Muzeum Narodowego w Warszawie
Karolina Tabak
Karolina Tabak
