# Supplementary material for: Evolution of European bison image and its implications for current species conservation
Source: PLoS One. 2023 Jan 31;18(1):e0281113. doi: 10.1371/journal.pone.0281113 (PMC9888683; doi:10.1371/journal.pone.0281113)
Supplement: S3 File — (DOCX) [file pone.0281113.s004.docx]

**Copyright explanations for figures in the manuscript**

All illustrations used for analysis were made prior to 1900 (except of European bison photograph from Wikipedia page on European bison, explicitly shared under CC BY 4.0 license). Authors of the youngest illustrations died in 1902 (Beckmann), 1915 (Lydekker), 1918 (Friese), 1911 (Hayek), 1923 (Brochocki) – all of them over (or almost over) 100 years ago.

As for specific illustrations:

1. In answer to my request, the British Museum allowed only to publish the images under a Creative Commons Attribution-NonCommercial-ShareAlike 4.0 International (CC BY-NC-SA 4.0) license. Therefore, I decided to remove the image from Fig 1, replacing it with hand-drawn outline representing the original Durer’s work and explained it both in Methods section and in Appendix 1. Additionally, Appendix 1 has a link at which readers can view the original image. This allowed to maintain the manuscript integrity (removing one image would alter the results of the analysis).

2. Website of the Rijksmuseum (http://hdl.handle.net/10934/RM0001.COLLECT.29680) states that the image is in public domain (CC0 1.0 Universal (CC0 1.0) Public Domain Dedication)

3. Website of the Lower Silesian Digital Library (https://dbc.wroc.pl/dlibra/publication/6046/edition/5639?language=en) states that the entire book from which the illustration is taken is in public domain

4. Biodiversity Library (https://www.biodiversitylibrary.org/bibliography/125499) states at copyright status: “Copyright Status: NOT_IN_COPYRIGHT”

5. E-manuscripta webpage (dx.doi.org/10.7891/e-manuscripta-92207) states in “Terms of Use”: Public Domain Mark. Available for free download. In the event of reproduction or exploitation of the document or parts thereof, the holding institution asks you, in accordance with usual scientific practices, to provide:

information;

indication of sources including details of the author, location and persistent identifier;

dispatch of a voucher copy and/or notification of the internet address of your digital offering into which you have integrated the document or parts thereof

6. Website of the Rijksmuseum (http://hdl.handle.net/10934/RM0001.COLLECT.168100) states that the image is in public domain (CC0 1.0 Universal (CC0 1.0) Public Domain Dedication)

7. Website of the Rijksmuseum (http://hdl.handle.net/10934/RM0001.COLLECT.673390) states that the image is in public domain (CC0 1.0 Universal (CC0 1.0) Public Domain Dedication)

8. Polona website (https://polona.pl/item/historiae-naturalis-de-quadrupedibus-libri-cum-aeneis-figuris,MTg1NDM1NDg/80/#item) states the image is in Public Domain.

9. Website of the Rijksmuseum (http://hdl.handle.net/10934/RM0001.COLLECT.336499) states that the image is in public domain (CC0 1.0 Universal (CC0 1.0) Public Domain Dedication)

10. Wikipedia page hosting this image (https://commons.wikimedia.org/wiki/File:Muentz_Bison_Gilibert.jpg) states it is public domain.

11. In a e-mail from the Department of Drawings of Warsaw University Library, Royal Collection, I received the following information:

„Szanowny Panie Doktorze,

przytoczony przez Pana wizerunek akwareli J. Potockiego (Inw.zb.d. 10206) znajduje się w domenie publicznej i z tego względu nie jest objęty autorskimi prawami majątkowymi, można nim zatem dysponować wedle własnego uznania. Z tego względu nie mamy uprawnień, aby wydawać tego rodzaju pozwolenie na jego publikację.

Z wyrazami szacunku

Marcin Falkowski

Gabinet Rycin

Biblioteki Uniwersyteckiej w Warszawie

ul. Dobra 56/66,00-312 Warszawa

+48 22 5525830”

translation:

“Dear Dr,

the image of the watercolor by J. Potocki (inv.zb.d. 10206) you cited is in the public domain and therefore is not covered by copyright, so you can use it at your own discretion. Therefore, we are not authorized to issue this kind of permission for its publication.

Best regards

Marcin Falkowski

The Reproduction Room

of the University Library of Warsaw

56/66 Dobra Street,00-312 Warsaw

+48 22 5525830”

12. Website of the Library of University of Heidelberg where the scan of the image is hosted (https://digi.ub.uni-heidelberg.de/diglit/bertuch1795bd2/0042) states it is in Public Domain.

13. Website of the Biodiversity Heritage library hosting the scan of Buffon’s book (https://www.archive.org/download/buffonsnaturalhi08buff/page/n30_w312) states it is in Public Domain (Public Domain = Current Year – 95, https://about.biodiversitylibrary.org/help/copyright-and-reuse/#pubdomain)

14. Website of Elblag Digital Library holding the scan of the issue (https://dlibra.bibliotekaelblaska.pl/dlibra/publication/48096/edition/45288/content) states it is in Public Domain.

15. Website of Silesian Digital Library hosting the book (https://www.sbc.org.pl/dlibra/publication/93016/edition/87770/content) states it is in Public Domain.

16. Website of Polona digital library hosting the image (https://polona.pl/item/zubrzyca,MTE5MTU2Mzkz/0/#info:metadata) states it is in Public Domain

17. Website of the Biodiversity Heritage library hosting the scan of Brincken’s book (https://www.biodiversitylibrary.org/bibliography/70897) states it is in Public Domain.

18. Website of Google Books hosting the scan of Eichwald’s book (https://books.google.pl/books?id=_3VNAAAAcAAJ) states it is in Public Domain.

19. Website of the Digital Repository of Scientific Institutes hosting the scan of Jarocki’s book (https://rcin.org.pl/dlibra/publication/156546/edition/134060/content) states it is in Public Domain.

20. Website of Google Books hosting the scan of Barlow’s book (https://books.google.pl/books?id=__heAAAAcAAJ) states it is in Public Domain.

21. Website of the Biodiversity Heritage library hosting the scan of Cuvier’s book (https://www.biodiversitylibrary.org/item/198514#page/9/mode/1up) states it is in Public Domain (Public Domain = Current Year – 95, https://about.biodiversitylibrary.org/help/copyright-and-reuse/#pubdomain).

22. Website of Lower Silesian Digital Library hosting the scan of Chodźko’s book (https://www.dbc.wroc.pl/dlibra/publication/6508/edition/6031?language=en) states it is in Public Domain.

23. Permission to publish the photograph of Ruseckas K (1843) Dogs attacking an European bison. Lithuanian National Museum of Art T-1498 is attached as pdf file.

24. Website of the Library of University of Heidelberg hosting the image (https://digi.ub.uni-heidelberg.de/diglit/schreber1844tafelbd2/0272/image) states it is in Public Domain.

25. Website of Google Books hosting the scan of the image (https://books.google.pl/books?id=7q5LAAAAcAAJ) states it is in Public Domain.

26. Website of the Biodiversity Heritage library hosting the scan of the illustration (https://www.biodiversitylibrary.org/item/54554#page/161/mode/1up) states it is in Public Domain.

27. Website of the Biodiversity Heritage library hosting the scan of the book (https://www.biodiversitylibrary.org/item/56383) states it is in Public Domain.

28. Website of Polona library hosting the scan of the book (https://polona.pl/item/opowiadania-ojca-obejmujace-historya-naturalna-jeografia-historya-polska-i-starozytna,MTA3MTY2NA/135/#item) states it is in Public Domain.

29. The image was directly scanned by authors from the book published in 1862. No information about copyright is available. Author of the image, Michaly Zichy, died in 1906 (https://en.wikipedia.org/wiki/Mih%C3%A1ly_Zichy)

30. Website of the Digital Library of the University of Łódź (https://bcul.lib.uni.lodz.pl/dlibra/publication/1512/edition/1174/content) hosting the image states it is in Public Domain.

31. Website of Google Books, hosting the scan of the book (https://books.google.pl/books?id=H1NVAAAAcAAJ) states it is out of copyright.

32. Website of the Biodiversity Heritage library hosting the scan of the book (https://www.biodiversitylibrary.org/item/16032#page/424/mode/1up) states it is in Public Domain.

33. Website of Polona library hosting the scan of the book (https://polona.pl/item/obrazki-z-zycia-zwierzat-bobr,MzkzOTYyNDY/97/#item) states it is in Public Domain.

34. Website of the Digital Library of the University of Łódź hosting the image (https://bcul.lib.uni.lodz.pl/dlibra/publication/421/edition/272/content) states it is in Public Domain.

35. Website of Kujawsko-Pomorska Digital Library hosting the scan of the book (https://kpbc.umk.pl/dlibra/publication/30919/edition/39801/content) states it is in Public Domain.

36. Website of Rijksmuseum Twenthe hosting the downloadable scan of the image (https://collectie.rijksmuseumtwenthe.nl/zoeken-in-de-collectie/detail/id/2dc5dfd6-e1db-55c8-bc3a-8ba016817df7) states “If an image has a download button, you’re welcome to use it for any purpose you like”.

37. Website of the Biodiversity Heritage library hosting the scan of the book (https://www.biodiversitylibrary.org/item/36741#page/97/mode/1up) states it is in Public Domain.

38. I received the permission to publish the figure [Beckmann L (before 1900) Die Auerstiere im Zoologischen Garten zu Köln. Nach der Natur gezeichnet von L. Beckmann. National Museum in Warsaw, nr inw. Gr.Pol.28499] from the National Museum in Warsaw (attached as pdf file).
